# Evaluability assessment: An application in a complex community improvement setting

**DOI:** 10.1177/1356389019852126

**Published:** 2019-06-02

**Authors:** Richard Brunner, Peter Craig, Nick Watson

**Affiliations:** University of Glasgow, UK; University of Glasgow, UK; University of Glasgow, UK

**Keywords:** area-based, collaboration, evaluability assessment, public services, theory of change

## Abstract

Evaluation is essential to understand whether and how policies and other interventions work, why they sometimes fail, and whether they represent a good use of resources. Evaluability assessment (EA) is a means of collaboratively planning and designing evaluations, seeking to ensure they generate relevant and robust evidence that supports decision-making and contributes to the wider evidence base. This article reports on the context, the process undertaken and evidence from participants in an EA facilitated with public service workers involved in implementing a complex, area-based community improvement initiative. This is a novel context in which to conduct an EA. We show how the process allows practitioners at all levels to identify activities for evaluation and co-produce the theory of change developed through the EA. This enables evaluation recommendations to be developed that are relevant to the implementation of the programme, and which take account of available data and resources for evaluation.

## Introduction

Governments across the advantaged world have increasingly recognised that public services need to be reorganised and delivered through collaborative approaches if they are to enhance their affordability and effectiveness (Hughes, 1998; Pollitt and Bouckaert, 2000). The process of evaluation is central to the public service reform process (see, for example, Halpern, 2016), yet developing reliable evaluation methodologies has not been high on the public service reform agenda (Boyne et al., 2004; Hanson and Jones, 2017; Osborne and McLaughlin, 2002). Attempts at evaluation have tended to lack a clear theoretical basis and operate within a poorly defined theory of change (Pollitt, 2000). Some successes have been claimed for collaborative approaches to evaluation (O’Sullivan and D’Agostino, 2002), but problems are also evident. For example, in an evaluation of Health Action Zones in England, Sullivan et al. (2002) found that resource constraints made such approaches difficult and prevented the development of a theory of change for the programme. In this article, we report on our experience of using evaluability assessment (EA) as way of establishing a collaborative approach to evaluation.

EA is an approach to evaluation planning in which researchers and intervention stakeholders work in partnership to co-produce a theory of change. Through bringing all those involved in an intervention together, EA aims to develop an evaluation approach based on a shared understanding and that takes account of local issues to deliver useful information for decision makers. The article describes how EA was used to develop and recommend an evaluation framework for *Thriving Places* (TP), an area-based approach to tackling deprivation in Glasgow, Scotland. To our knowledge, this is the first time that an EA has been conducted in this type of multi-agency environment. Previous EAs have tended to focus on initiatives where the outcomes are highly focused. Using contemporaneous data and ex-post interviews with a sample of participants we show how EA enabled public service workers to collectively define the aims and outcomes of the initiative, clarify their understanding of its principles and outcomes, identify activities, and produce a workable evaluation framework. The article identifies limits to the process.

In section ‘Evaluability assessment’ we introduce our approach to EA, while the next section describes *Thriving Places*. Section ‘Method’ sets out the methods we used to conduct the EA and to gather participants’ views. Section ‘Findings: co-producing an EA for TP’ presents our findings and Section ‘Discussion and conclusion’ discusses the implications of the findings for future EAs.

## Evaluability Assessment

EA is a systematic approach to planning evaluation projects. It involves structured engagement with stakeholders to clarify intervention goals and how they are expected to be achieved, development and evaluation of a logic model or theory of change, identification of existing data sources, and provision of advice on whether an evaluation can be carried out at reasonable cost or whether further development work on the intervention should be completed first. EA involves time and effort, but the investment is generally small relative to the costs of a full-scale evaluation. Committing resources to an EA should be worthwhile if it leads to a better understanding of how interventions work, more realistic expectations of what evaluation can and cannot deliver, and more effective and efficient evaluation designs.

EA was originally developed in the United States in the 1970s as a way of reducing the waste associated with evaluating social programmes that were so poorly designed or implemented that no impact could realistically be expected (Van Voorhis and Brown, 1997; Wholey, 1987). Legislation requiring federal bodies to report on performance encouraged the use of EA from the 1990s onwards (Leviton et al., 2010), and EA is now standard practice in some agencies such as the Centres for Disease Control (Losby et al., 2015). Outside the United States, EA has been most widely used to plan the evaluation of international development projects, but has recently attracted interest from evaluators working in public health (Ogilvie et al., 2011; Petticrew et al., 2013). Its use in the evaluation of public service reform at a local level is still in development. The published literature on EA is fragmented, consisting predominantly of grey literature reports, though there is a small but growing body of peer-reviewed papers (Trevisan, 2007), and a number of useful guidance documents and critical reflections (Davies, 2013; Davies and Payne, 2015; Dunn, 2008; Leviton et al., 2010; Peersman et al., 2015).

Davies (2013) has identified a number of core elements from a review of existing guidance. They include the following:

1. Defining the boundaries of the intervention that is to be evaluated, identifying relevant stakeholders and agreeing on expected outputs;
2. Identifying the resources available, such as relevant documents, information systems and datasets;
3. Engaging with stakeholders, to identify their understandings of the intervention and their expectations of an evaluation;
4. Developing conclusions and making recommendations for the design or logic of the intervention, the development of information systems and possible evaluation designs;
5. Feeding back findings and conclusions to stakeholders.

Most guidance on EA emphasises the need for flexibility so that the process can be tailored to the resources available, and to ensure that the effort invested in the EA is proportionate to the cost and complexity of the intervention. We have developed an approach that brings together these elements into a series of workshop-style meetings with stakeholders, conducted over a period of 3–4 months, culminating in the presentation of recommendations based on an appraisal of evaluation options (Craig and Campbell, 2015). The process is led by researchers, but relevant policy-makers, practitioners and others involved in delivering the intervention are engaged at all stages.

**Figure 1.**
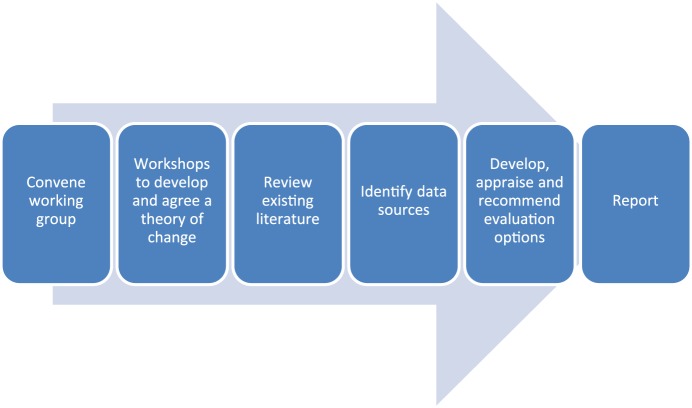
Stages of an evaluability assessment.

For simplicity, the process is presented in Figure 1 as a sequence of steps, which in practice will overlap. The amount of time and effort needed at each stage will vary from one EA to the next, depending on factors such as the strength of the existing evidence base, availability of routinely collected data, complexity or degree of development of the intervention and number of key stakeholders. The focus of the early meetings is on developing a theory of change. In later meetings, the emphasis shifts towards confirming the theory as a valid model of stakeholders’ expectations of how the intervention works, and discussing alternative approaches to evaluation. The output is a published report, presenting the theory of change, setting out an appraisal of the evaluation options and recommending an option that is feasible, and achievable with the resources available.

EAs have recently been conducted of a number of social and public health interventions in Scotland (Beaton et al., 2015; Myers et al., 2017; Wimbush et al., 2015), and have led to the commissioning of evaluations. We follow Leviton et al. (2010) in defining EA as a pre-evaluation activity. It is important to be clear about where the EA process ends, and evaluation begins, because there is no guarantee that an EA will lead to a recommendation in favour of conducting an evaluation. Instead the process may reveal that evaluation would be premature, given the state of development of the intervention, or disproportionately costly, given the availability of data, size of effects expected or the difficulty of designing a high-quality evaluation study (Davies and Payne, 2015). The involvement of stakeholders in the process should help to ensure that the basis for the recommendation is well understood.

## Thriving Places

Scotland has a statutory system of local governance in which public services and other key partners within the 32 local authority boundaries collectively constitute a Community Planning Partnership (CPP); (Local Government in Scotland Act 2003). CPPs are a central feature of public service reform in Scotland, intended to ensure that local public services, the third sector, community and private sector develop a shared vision for their area, and work in partnership to implement this (Sinclair, 2008). Since 2008, each CPP has been required by the Scottish Government to periodically produce a single outcome agreement (SOA) setting out the priority outcomes for their area, and how the CPP will work towards achieving them. The Community Empowerment (Scotland) Act 2015 has now replaced the 2003 Act, making a number of changes to the framework for CPPs, including the supercession of SOAs with local outcome improvement plans (LOIP).

In its 2013 SOA, setting out the shared priorities for public services over the next decade Glasgow CPP used Scottish Index of Multiple Deprivation (SIMD) trend data to identify nine geographical areas, each of approximately 10,000 people, with the highest persistent deprivation relative to other parts of the city (Glasgow CPP, 2013). It argued that in these areas the issues shaping and surrounding persistent deprivation were complex and multilayered and called for ‘an approach that will make best use of the full range of resources and assets of the CPP to deliver better outcomes for these neighbourhoods’ (Glasgow CPP, 2013: 29). A decade-long commitment to a place-based approach, TP, was proposed. Three of these areas embarked on their TP programme in 2014–2015, with three further areas adopting the model from late-2016.

The 2013 SOA proposed a set of principles for public services operating in the TP areas (Glasgow CPP, 2013), changing the way in which resources are allocated if required; a long-term focus on partnership working; joint working at a very local community level; a focus on community capacity building; a focus on co-production between communities and organisations; and intensive activity to build social capital and empower communities, including making the most of assets. TP incorporated an ethic of relative autonomy – that a single programme model, set of solutions or configuration of professionals would not suit all places, and that each area should therefore develop its own path to improving its outcomes (Glasgow CPP, 2013).

Although TP is ostensibly a place-based intervention, the activities and outcomes are predominantly people-based (Kintrea, 2007). The focus is on improved ways of working including co-production, service integration and partnership working. The only funding specifically allocated for TP in 2015–2016 was £35,000 towards staff and development costs to each TP area. The funds were awarded to an appointed anchor organisation (Henderson and Escobar, 2018) in each area to pay for a single community connector post. Public services and third-sector organisations in each area were expected to devise and implement a wide array of local activities and develop new ways of working with communities. Strategic staff with city-wide roles in public services and those with strategic roles at the centre of the CPP were also expected to change the way they work and to ‘bend the spend’ towards the most disadvantaged communities in the city.

The 10-year outcomes proposed for each of the TP areas were as follows (Glasgow CPP, 2013: 31–2):

- The creation of more resilient, sustainable communities which are stable, thriving and growing, and people are proud to live in;
- Communities have more aspiration and influence over the planning and commissioning of local services by CPP partners;
- Communities across the city which would work in partnership with CPP bodies to develop services for local residents;
- Levels of demand for particular local services shift (both up and down) as both needs and awareness levels change.

In late 2015, 1 year into the 10-year programme, and with three TP areas already underway, we were asked by Glasgow CPP to work with them to help establish an evaluation framework for TP. At the time, the CPP had not developed a clearly articulated theory of change for TP. The problems associated with the evaluation of area-based initiatives are well documented (Gibbons et al., 2014). Data at the appropriate spatial scale are often unavailable, the boundaries of the initiative may not coincide with administrative boundaries and few surveys are large enough to provide usefully precise data for small areas. Where data are available, proving causality is challenging and it is often difficult to determine what would have happened in an area if an initiative had not been implemented (Gibbons and Overman, 2012). EA, we felt offered the best prospects for developing a framework for evaluation. In the next section, we document how we went about the EA process.

## Method

This article is based on two data sources. First, our contemporaneous notes collected while conducting the EA for TP, and, second, data drawn from retrospective telephone interviews with a purposive sample of participants in the process. Because this was the first EA we had conducted of a community-based intervention with such a broad range of stakeholders, we wanted to gain an understanding of what participants thought of the process and the impact it had on them.

### The EA process, activities and resources for evaluation

In 2015–2016, we facilitated a series of workshops with a range of officers involved with TP. Prior to the first workshop, we read through all the key policy documents to develop a model that represented our interpretation of the key principles and 10-year outcomes for TP – its draft theory of change. These were presented to the first set of workshops and were used to stimulate dialogue among participants. We organised two initial half-day workshops, one aimed at those who had a strategic role in the development of TP across the city, and one for those with an existing operational role in the three extant TP areas. We proposed that the two groups be facilitated separately so that any distinctions between strategic and operational understandings of TP could be drawn out. This allowed us to probe the accuracy of our interpretation but to also determine how unified the understanding of the principles and outcomes of TP was across partners working at different levels. In all, 7 strategic and 13 operational TP practitioners attended these workshops. There was a strong alignment in their understanding of the aims and values of TP and we made only minor amendments to our draft theory of change.

**Figure 2.**
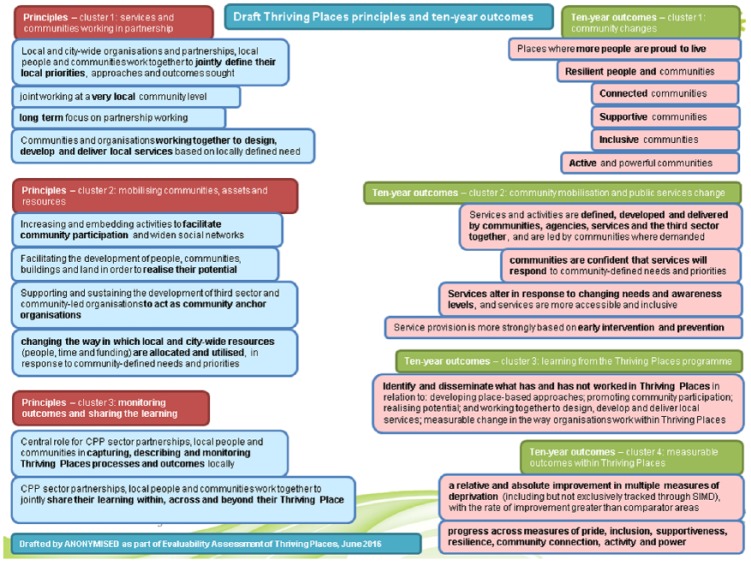
Final principles and 10-year outcomes diagram for Thriving Places.

EA is an iterative process and is able to be responsive to circumstances. Due to the strong consensus that emerged at the first workshops, we changed our plan and brought the two groups together for Workshop Two, with 20 officers attending. At this workshop the theory of change was further refined, and in the second half of the workshop we turned our attention to developing the evaluation recommendation. Prior to the workshop, we asked participants to identify exemplar activities from their work that they felt were central to TP. We wanted the EA to reflect the actual work on the ground from the perspective of those actually doing it in the three TP areas. In all, 16 activities were provided by a diversity of services and organisations. These included strategic activities at the level of the CPP such as realigning staff time to support TP, ‘bending spend’, and aligning commissioned services to focus on the TP areas. Local activities included initiatives aimed at promoting adult learning and digital inclusion, occupational therapy support for employability, community health interventions such as singing and smoke-free services, interventions aimed at promoting participation such as a community conversations project, a ‘talking garden’, and participatory budgeting and activities to improve local data collection. After the workshop, we mapped these activities onto the final Principles and Ten-Year Outcomes diagram (Figure 2). Every TP principle, except *Supporting and sustaining the development of third sector and community-led organisations to act as community anchor organisations* were covered by these activities. All of the Ten-Year Outcomes were covered although outcome clusters three and four, focused on learning and measurable outcomes were, understandably, more sparse at this early stage of TP. At Workshop Three we presented the final Principles and Outcomes diagram together with our recommendation for evaluation (see section ‘Findings: co-producing an EA for TP’).

Participants at the workshops included officers from across the range of public services, reflecting the complex multi-agency context for TP. Agencies represented included health, housing, culture, strategy, fire, policing, community development, education, enterprise and the third sector. Seniority also varied, including one elected member, strategic managers, middle managers and operational officers across the services. Workshop participants did not all know each other beforehand. Inconsistency of workshop attendance was experienced with officers replacing each other, officers missing workshops, and some services attending only one or two workshops.

### Retrospective interviews

Due to the diffuse character of this EA, in order to improve our capture of the range of potential experiences and outcomes, we conducted eight interviews in late 2016 with a sample of participants, selected to bring together a mix of those with strategic and operational involvement in TP, and those in different services. These were audio-recorded and transcribed for subsequent analysis. The interviews asked about views on the EA process, its outcomes and whether involvement in the EA process had impacted on their thinking about TP or about evaluation of TP. We independently read the interview transcripts and our own field notes and coded them manually, looking for emerging themes. We then met together to produce a coding framework as a basis for analysis, building on themes present in the extant EA literature and themes emerging from the data. Through this iterative process, we developed more detailed coding as themes and sub-themes emerged. After the initial round of eight interviews we reflected on the key findings and felt that we had reached ‘thematic data saturation’, with no more new patterns or themes emerging (O’Reilly and Parker, 2013). Ethical approval for the research was obtained from our university ethics panel.

## Findings: co-producing an EA for TP

This section describes findings from the key elements of the EA process. It draws on our contemporaneous notes and on participants’ interview responses about the EA workshop process and the final theory of change diagram.

### The EA workshops

One of the major concerns of both of the first workshops was the need to make sure that the documentation that emerged from this process was easily understandable by all. Using terms to describe the work in a language that avoided jargon was seen as essential by participants. Terms such as co-production, asset-based working and other similar technical expressions were rejected. Instead, participants proposed replacements such as ‘jointly define their local priorities’ and ‘working together to design, develop and deliver new services’. The CPP was also keen to ensure that individual TP principles and outcomes should not be interpreted as being the sole responsibility of a single public service. Therefore, terms such as ‘healthy communities’ or ‘safe communities’ were rejected.

Between the first and second workshops the most contested interpretations of TP included whether, in terms of principles, TP was focused on ‘changing the way in which local and city-wide resources are allocated and utilised’ in response to either ‘community needs’ or ‘community-defined priorities’, and whether, in terms of outcomes, TP was about achieving ‘more resilient’ or ‘less vulnerable’ communities. The final agreed wordings for these (‘community-defined needs and priorities’ and ‘more resilient people and communities’) can be seen in the final theory of change diagram (Figure 2). The facilitated EA process sought to offer participants the space to air differences and reach a consensus across a range of issues. Without this constructive engagement, any varying interpretations of TP would have remained unexamined and ambiguous, in turn constraining programme implementation and programme evaluation.

Both during and after the process, participants felt that the EA approach was distinct from normal meetings associated with work with the CPP. One participant felt that it was *‘*more like a learning process than a planning process’. An operational officer noted: *‘*the whole, sort of, community planning meeting structures are very formal, a bit dictatorial, a bit hierarchical’; in contrast the EA was *‘. .* . very welcoming, well put together, very well facilitated’. A strategic officer suggested that the collaborative EA process offered a means to ameliorate traditional anxieties at strategic level:

. . . it felt more inclusive than some of the other community planning processes . . . Sometimes it feels like . . . there’s challenge and dynamics just by the nature of community planning and what happens centrally and what happens locally and . . . where the power sits . . . So, it actually felt like a bit of work . . . that we were able to just get on [with] without worrying too much about what people might think of it at the end . . .

One of the advantages of EA became apparent early in the process: it offered the potential for changing both the direction and the ownership not just of the evaluation methodology but also the principles and intended outcomes of the 10-year TP programme itself. The senior officers of the CPP had agreed to open up a core element of their professional jurisdiction – strategic planning, monitoring and evaluation – to the influence of operational officers and others; a new experience for all involved. It helped to move TP from a top-down proposition, written by senior officers for a strategic document (Glasgow CPP, 2013) to a programme co-produced by strategic and operational officers working together. Through collective clarifying and consensus-building a common understanding of TP emerged:

I think that was really good for trying to get us in a position across the city to say that we agreed with what those principles were and of the language used and that they were representative of what the different agencies were trying to achieve.

Evaluation could focus on issues that matter to those operationally involved in the programme, not just those with a strategic responsibility.

However, this opening up of the process was not without its challenges and part of our role was to manage any tensions that arose. We did this by emphasising throughout that the evaluation recommendation emerging from the EA would complement rather than replace, the summative Performance Management Framework already developed by the CPP. The strategic staff sought to manage this tension by insisting on sight of our draft report prior to the final EA workshop at which the evaluation recommendation was made to the full group. At one point we had to hold an emergency meeting with senior management to try and allay their fears.

We thought that we had pushed EA a long way in terms of collaboration. However, as several participants highlighted, a key constituent had been absent from the process. Although TP was about community empowerment, community members were not part of the EA process. A senior officer noted:

. . . if the process had involved a different group . . . such as community resident groups, residents themselves, they might have come up with a different set of outcomes and principles . . .

However, some participants suggested that including community groups would have been a risk at this stage:

. . . I remember some of the discussion was around just a word or whether a word [in the draft Theory of Change] was appropriate . . . around what some people would just see as jargon. So I think we would have frightened a lot of people away . . . and made them think that we’re completely wasting our time or their time, ‘cause it wouldn’t have seemed relevant.

Other workers saw this as part of a developmental tension inherent to taking a community development approach to TP:

The people that were missing that you really want, it’s difficult to achieve, which is representatives of the communities themselves. So . . . this is, if you like, a top down approach . . . trying to make it bottom up . . .

That criticism notwithstanding, the early workshops created a distinctive, deliberative approach to evaluation planning, and through this facilitation a collective understanding of TP developed across involved strategic and operational officers.

### The theory of change diagram

The final principles and outcomes diagram (Figure 2) sought to offer all strategic and operational participants a unified representation of the TP outcomes they were commonly seeking to achieve. Through the EA workshops, we thematically grouped the key principles and 10-year outcomes of TP into clusters, checking throughout the process whether the themes and terminology made sense to participants, and finally testing that the exemplar TP activities provided by participants also correlated with the clusters. The three clusters of principles were services and communities working in partnership; mobilising communities, assets and resources; monitoring outcomes and sharing the learning. The four clusters of 10-year outcomes were community changes; community mobilisation and public services change; learning from the TP programme; measurable outcomes within TP.

By bringing together the principles and clarifying the outcomes and presenting these diagrammatically we were able to create a co-owned, unified understanding of TP. We were repeatedly told that the diagram accurately reflected the aims and outcomes of TP, for example:

. . . it’s a pretty well simplified way of expressing what we’re trying to achieve . . . Now obviously it’s the product of compromise between a whole range of different takes on it. But I think at the end of the day it summarises things pretty well. What’s particularly important about it is it’s a useful way of explaining to all the staff and . . . in due course to relating to participants what it is that we’re trying to do.

While creating a shared account of how an initiative is expected to achieve change is one of the most important outcomes of an EA (Leviton et al., 2010: 219), here we have been able to demonstrate how this principle applies even to programmes that are as complex and multiagency as this one.

Visually laying out the model and using clear, precise terminology was seen as being particularly useful:

I think it’s incredibly helpful, having something visual is good, the language is pretty clear and straightforward, and I think it’s a really, really, helpful tool. I think there’s caveats in terms of how it’s used, and there’s probably some just cautions in terms of how and when you might use it and being clear to people how it was arrived at. But, I think as an actual working document it’s incredibly helpful.

The diagram, we were told, had been used beyond the evaluation process, including for strategic and practical planning by officers and community organisations. It had become a tool for evaluation in its own right, with one participant using it as a tool for self-evaluation and for development of her staff: ‘. . . we should be saying, to what extent do we think we’re contributing towards these principles and towards these outcomes?’ It had also been used to explain to other officers what TP was about, and had been applied strategically to inform the development of Locality Plans in the city.^1^

### The evaluation recommendation

We recommended that the initiative adopt a radically different approach to evaluation than that originally envisaged by the strategic managers of TP. The original evaluation plan was for a summative Performance Management Framework (PMF) drawing on a range of routinely collected indicators. While these indicators would provide useful information about the context within which TP was operating, our analysis suggested that it would not be able to evidence the type of change TP would be able to deliver. We were particularly concerned about the ‘power’ of the survey data to detect an effect of the size that might be expected from TP. In order for the changes to be detectable, TP would have to achieve improvements in indicators of community well-being that were larger than the changes for similar indicators seen in the far more substantially resourced New Deal for Communities in England (Batty et al., 2010). Given the reach of TP activities (i.e., the proportion of the TP population directly involved), it appeared unlikely that such large changes would be observed. Extra investment in quantitative data collection via population surveys therefore appeared to be of questionable value. This EA finding and recommendation was the cause of the tension noted above.

Instead, we recommended that, given the volume, character and reach of the activities associated with TP proposed at Workshop Two, in addition to the proposed PMF, evaluation effort should be invested in formative qualitative evaluation. We recommended a structured series of case studies with a purposive sample of TP activities, selected to encompass (1) the three existing TP areas, (2) a range of levels of intervention (single area, multiple area, and Glasgow-wide) and (3) a range of 10-year outcome clusters (Brunner et al., 2017). If systematically explored through a case study approach this held the potential to provide short- to medium-term indications of the types of intervention that fulfilled the principles and expectations of TP, alongside examples of those that were less successful. Services and partnerships across both existing and emergent TP areas could use this evidence to adapt their approaches and activities. Our recommended approach would require high-level research and facilitation skills, including collaborating with staff, service users and citizens.

These recommendations were based on the facts and values expressed by participants in the workshops and on the exemplar TP activities supplied to us by participants at Workshop Two. They took into account the CPP’s planned summative evaluation through the PMF and were modified to meet the current economic context of austerity, in which large-scale resources were not available for evaluation. However, the recommendation also took account of the necessary research skills and methodological rigour, meaning that some resources would be required.

In the interviews, both operational and strategically focused participants said that they were not surprised by the formative case study evaluation recommendation. A strategic officer said:

. . . in terms of the decision to do the case study, yes, I absolutely get that, because . . . there are a lot of intangibles here in terms of how you capture the progress made . . . So, from that point of view, yes, had to take a case study approach.

An operational officer noted:

I think it’s probably going to be one of the most powerful evaluation methods we have, because there was a difficulty in taking things from a piece of paper to real life subjects and we often don’t have any, kind of, [way] to connect them.’

The recommendation tended to reinforce professional understanding that TP is qualitative and emergent:

I think because of the nature of what I think we’re aiming to do in Thriving Places, a lot of which is . . . very organic, very flexible, not necessarily easily measurable, would be entirely different, we have got different scale, pace, reach, etcetera, so, aye, trying to manage that in a kind of systematic number crunching way is very difficult.

This reflected how some participants already evaluated elements of their work: ‘I think in the [anonymised] service we use a lot of case studies, you know, to make recommendations with other key partners, so it wasn’t anything new’.

The consensus for case study evaluation that emerged at the end of the process was striking, and further establishes the value of EA in this context. At the outset of this process and for much of its duration strategic officers were strongly wedded to the idea of summative secondary data analysis as the source of evaluation. Through working with those involved we were able to explain how and why this would not provide effective evaluation of impact, and were able to facilitate the co-production of a collaborative evaluation proposal that would deliver effective information which officers at all levels understood and supported.

### Taking the EA forward

Although, as noted above, some participants had used the Principles and Outcomes diagram in a variety of local contexts, other participants were unclear about ‘next steps’ following the EA process. Several were waiting for leadership on dissemination of the diagram and advocacy for the evaluation recommendation. Reverting to traditional ‘command-and-control’ models, and away from the collaborative governance processes facilitated through the EA (Huxham et al., 2000), some interviewees expected the theory of change diagram to be formally approved by the CPP hierarchy:

. . . it was my understanding that they were going for approval to the CPP Strategic Board but we’ve never had communication about whether they have been approved . . . Again with that recommendation around case studies, we’ve never had any kind of follow-on from that . . . There’s never been any feedback locally, like wider, to wider partners than those involved. So . . . the loop hasn’t been closed yet.

However, neither of the strategic interviewees expressed clarity about their role in leading the diagram dissemination or implementing the evaluation recommendation, one noting,

I don’t think I should have ownership, I’m not the person that should have ownership. That should be a question that’s directed at people directly who have a stake in each of the Thriving Places.

This reflects wider evidence of ambiguities of leadership in CPPs (Sinclair, 2008). Here, the CPP felt that its role was not to direct and that, for TP to succeed, decisions had to be made at the local level. This does not, however, appear to have been communicated with sufficient clarity to those involved with delivering the services at the local level.

## Discussion and conclusion

Co-producing an EA in this semi-autonomous, multi-service, multi-site context for a programme seeking wide-ranging community improvement outcomes takes the approach beyond any previous applications of which we are aware. EA allowed us to bring together those strategically and operationally involved in this programme to develop both an evaluation recommendation with a clear methodological framework, and a consensual theory of change. We have shown how the collaborative and democratic evaluation propositions put forward by those such as O’Sullivan and D’Agostino (2002), Sullivan et al. (2002), Picciotto (2017) and Schwandt (2018) can be successfully operationalised in an encompassing, community improvement context.

In more traditional approaches, evaluation is about ‘looking backwards in order to better steer forward’ (Vedung, 2017: 2). Here, EA has allowed us to develop an evaluation method for a complex area-based intervention that not only looks forward but also shapes the policy and the initiative. Through facilitating collaboration between a wide range of strategic and operational officers from multiple public services and the third sector we have been able to ensure that the evaluation strategy determines the ‘merit, worth and value of things’ (Scriven, 1991: 1) that actually matter to those involved in managing and implementing the initiative. We have achieved this by getting people to work together and to openly discuss their own perspectives, so developing a shared understanding both of TP but also, importantly, the evaluation process itself.

The deliberative process we adopted through EA also moved evaluation from the periphery to the centre of the 10-year TP programme. EA shifted ownership of the principles, outcomes and evaluation of the programme away from being ‘top-down’ and managed at a senior level to, instead, being co-produced by strategic and front-line operational officers together. In this regard, this EA is an exemplar of the contemporary values and political context for public service reform (Christie Commission, 2011) and collaborative governance (Newman et al., 2004) in which power is directed towards enhancing collaborative activity (Huxham and Vangen, 2008, Chs. 9 & 10). The active facilitation and deliberative processes used in this example demonstrates how EA can actively support this transition. In practical terms too, this has twin benefits: the EA process is not only likely to minimise evaluation errors, but also to minimise the potential for practitioner misunderstandings and cross-purpose working in the field.

A theory of change is an essential component of any evaluation of complex, multi-partner, place-based urban initiatives such as TP. The programme outcomes are not predictable and as Moore et al. (2015) argue, an understanding of the causal assumptions that underpin the initiative are essential to any evaluation programme. Without such a framework, it is hard to see how an evidence base could emerge to inform future policy and practice (Craig et al., 2008). The theory of change has provided a pragmatic framework that describes the process of TP, and the evidence presented demonstrates ways in which the theory of change has gained traction as a resource in itself, influencing local practices beyond its originating purpose. This demonstrates how EA can impact beyond the formal evaluation recommendation. In this case too, the theory of change is aimed to be both flexible and to enable the identification of multiple causal pathways (De Silva et al., 2014). As TP develops and its activities and working practices evolve and are evaluated, there is capacity for the theory of change to evolve in response.

We expected that the CPP would drive forward the dissemination of the theory of change to officers beyond those directly involved in the EA, and that they would progress the evaluation recommendation. However, this leadership did not clearly emerge, leaving some officers uncertain of next steps. This highlights an important challenge for EA facilitators in comparable contexts: if strategic leaders do not take ownership of outcomes, even in ostensibly collaborative contexts, this could risk undermining or constraining the benefits of EA. At worst, not securing onwards leadership could result in perception of EA as wasteful of officer time. This suggests that gaining clarity on post-EA leadership by the commissioning body is as important in this context as clarifying the purpose and limits of EA itself, an extension of our EA model (Figure 1).

The EA process took several months to complete and was perceived by some participants as a resource intensive process. However, to sufficiently unpick potentially diverse initial concepts and theories of change, and varied interpretations of those concepts, and then re-present a clarified theory of change, followed by an evaluation recommendation linked to actual programme activities, is inherently complex. We doubt whether this could be done quicker without significant threat to the collaborative gains of the process, as noted above. Partnership working and co-production takes time (Cook, 2017; Cullingworth et al., 2018). Facilitating mutual understanding takes more time with a larger group, more so again in a multi-agency, multi-level context. The actual officer time participating in the EA workshops was 7.5 hr over 6 months, plus preparation of an exemplar activity; a little over one working day per officer to clarify the theory of change and evaluation process for a 10-year initiative. That this was sometimes perceived as time-consuming suggests how radical a change this way of working can be for public services. This was likely reinforced by pressures experienced in the context of austerity (Bynner, 2016; Hastings et al., 2015).

A significant constraint in conducting an EA in a wide-ranging and semi-autonomous community improvement programme is that not all affected practitioners can be directly involved. This risks self-selection of those already ethically committed to collaborative practices. This limitation may skew EA findings, although this was not suggested in our case. One of the critiques identified by involved practitioners, however, was the missing role of the community in this EA. While community involvement was not attempted, it is clear that involving citizens and community groups in an EA process alongside officers would require more time, more painstaking workshop facilitation, and even finer attention to terminology usage in response to the wider power and knowledge dynamics at play. Stronger planning would also be required to achieve buy-in to the process, maximise consistency of attendance, capitalise on the theory of change and implement the evaluation option.

This article has explored the operation of a collaborative approach to evaluation planning (EA) for a community improvement programme involving multiple public service partners, in which implementation is complex, and where outcomes may be unpredictable or hard to detect at the spatial scales typical of such initiatives. EA enables those managing and implementing the initiative to collaborate together in the evaluation planning process. It allows those involved at all levels with programmes to take account of cost, available data and available evidence, the collaborative EA process challenging traditional notions of evaluation of strategic programmes being the preserve of specialists, and reflecting contemporary principles of public service reform. Most of all it enables the evaluation outcomes selected to be relevant to the actual implementation of the programme, holding the potential to foster confidence in officers at all levels to collaboratively deliver and evaluate services based on this clarified understanding. In turn, this improves potential to achieve the real-world outcomes that involved public services collectively seek – in this case, to alter long-term trajectories of area-based multiple deprivation – and to know whether or not these are actually being achieved.

## Notes

1. Locality Plans are a statutory obligation from the Community Empowerment (Scotland) Act (2015) requiring Community Planning Partnerships to specify their plans to tackle problems in local areas of highest multiple deprivation.
